# Prevalence of Vancomycin-Resistant *Enterococcus* (VRE) in Companion Animals: The First Meta-Analysis and Systematic Review

**DOI:** 10.3390/antibiotics10020138

**Published:** 2021-01-31

**Authors:** Yusuf Wada, Ahmad Adebayo Irekeola, Engku Nur Syafirah E.A.R., Wardah Yusof, Lee Lih Huey, Suwaiba Ladan Muhammad, Azian Harun, Chan Yean Yean, Abdul Rahman Zaidah

**Affiliations:** 1 Department of Medical Microbiology and Parasitology, School of Medical Sciences, Universiti Sains Malaysia, Kubang Kerian 16150, Malaysia; wadayusuf34@gmail.com (Y.W.); profahmad007@yahoo.com (A.A.I.); engkunursyafirah@gmail.com (E.N.S.E.A.R.); wardahyusofyaacob@gmail.com (W.Y.); leelihhuey@gmail.com (L.L.H.); azian@usm.my (A.H.); yeancyn@yahoo.com (C.Y.Y.); 2 Department of Zoology, Faculty of Life Sciences, Ahmadu Bello University, Zaria 810107, Nigeria; 3 Microbiology Unit, Department of Biological Sciences, College of Natural and Applied Sciences, Summit University, Offa PMB 4412, Kwara State, Nigeria; 4 Department of Chemical Science, Federal University of Kashere, Gombe PMB 0182, Gombe State, Nigeria; suwaibana@gmail.com; 5 Hospital Universiti Sains Malaysia, Universiti Sains Malaysia, Kubang Kerian 16150, Malaysia

**Keywords:** *Enterococcus*, companion animals, vancomycin resistance, systematic review, meta-analysis

## Abstract

Antimicrobial resistance in companion animals is a major public health concern worldwide due to the animals’ zoonotic potential and ability to act as a reservoir for resistant genes. We report on the first use of meta-analysis and a systematic review to analyze the prevalence of vancomycin-resistant *Enterococcus* (VRE) in companion animals. Databases such as MedLib, PubMed, Web of Science, Scopus, and Google Scholar were searched. The information was extracted by two independent reviewers and the results were reviewed by a third. Two reviewers independently assessed the study protocol using the Preferred Reporting Items for Systematic Reviews and Meta-Analysis (PRISMA) checklist and the study quality using the Joanna Briggs Institute (JBI) critical appraisal checklist for prevalence data. OpenMeta analyst and comprehensive meta-analysis (CMA) were used for the meta-analysis. The random effect model was used, and publication bias was assessed using the Eggers test and funnel plot. Between-study heterogeneity was assessed, and the sources were analyzed using the leave-one-out meta-analysis, subgroup analysis and meta-regression. Twenty-two studies met the eligibility criteria, but because some studies reported the prevalence of VRE in more than one companion animal, they were considered as individual studies, and 35 studies were therefore added to the final meta-analysis. Sampling period of the included studies was from 1995–2018. Of the 4288 isolates tested in the included studies, 1241 were VRE. The pooled prevalence of VRE in companion animals was estimated at 14.6% (95% CI; 8.7–23.5%; *I^2^* = 97.10%; *p* < 0.001). Between-study variability was high (*t^2^* = 2.859; heterogeneity *I^2^* = 97.10% with heterogeneity chi-square (*Q*) = 1173.346, degrees of freedom (df) = 34, and *p* < 0.001). The funnel plot showed bias, which was confirmed by Eggers test (*t*-value = 3.97165; *p* = 0.00036), and estimates from the leave-one-out forest plot did not affect the pooled prevalence. Pooled prevalence of VRE in dogs and cats were 18.2% (CI = 9.4–32.5%) and 12.3%, CI = 3.8–33.1%), respectively. More studies were reported in Europe than in any other continent, with most studies using feces as the sample type and disc diffusion as the detection method. With the emergence of resistant strains, new antimicrobials are required in veterinary medicine.

## 1. Introduction

*Enterococcus* occurs in the intestinal flora of most humans and animals and is mainly found in habitats polluted by human and animal defecation [1,2]. *Enterococcus* has evolved as a significant nosocomial and community-acquired pathogen due to its ability to develop resistance to antimicrobials, especially vancomycin [3]. The last treatment choice, particularly for *Enterococcus*, is vancomycin [4]. Enterococcal-resistant strains were thought to have emerged as a result of human antimicrobials use and their use as growth promoters in the livestock industry [5]. A good example is the use of avoparcin, which has been used as a feed additive to support livestock growth in Europe, including Turkey [6,7,8]. Avoparcin and vancomycin belongs to the same glycopeptide family of antibiotics. Subsequently, this avoparcin was banned in Europe in 1997, but its effect still persists, resulting in the selection of vancomycin-resistant *Enterococcus* in farms and in animal gut [9].

There has been an evolution in the social role of companion animals, as their numbers have increased significantly over the past half a century [10]. Furthermore, the close relationship between pets and their owners has not only attracted more attention to their welfare, but also to the consequences of such proximity, which is the acquisition and transfer of genes that confer antibiotic resistance to bacteria [11,12,13].

Antimicrobial resistance in companion animals is therefore of major global concern to public health and would require a very subtle yet tough approach. Research on the epidemiology and the transmission of resistant bacteria between humans and animals and vice-versa has increased, and their zoonotic potential cannot be overlooked [14]. Dogs and cats have been reported as potential reservoirs for resistant genes [15]. Bacteria possessing these resistant genes colonize the gut of apparently healthy pets, and could pose a serious risk to humans by easily transmitting the resistant genes [16]. These pets may be colonized by human resistant bacteria, as antimicrobials are frequently used in human and companion animals for therapeutic and prophylactic purposes in everyday practice [13].

A meta-analysis and a systematic review were conducted in order to assess the risks and distribution of vancomycin-resistant *Enterococcus* (VRE) in companion animals globally with respect to their prevalence. This could help to provide basic information for the surveillance and formulation of appropriate and targeted policies for the control of antimicrobial resistance in companion animals. This is the first study to determine the pooled prevalence of VRE in companion animals worldwide.

## 2. Results

### 2.1. Search Results and Eligible Studies

**Figure 1:**
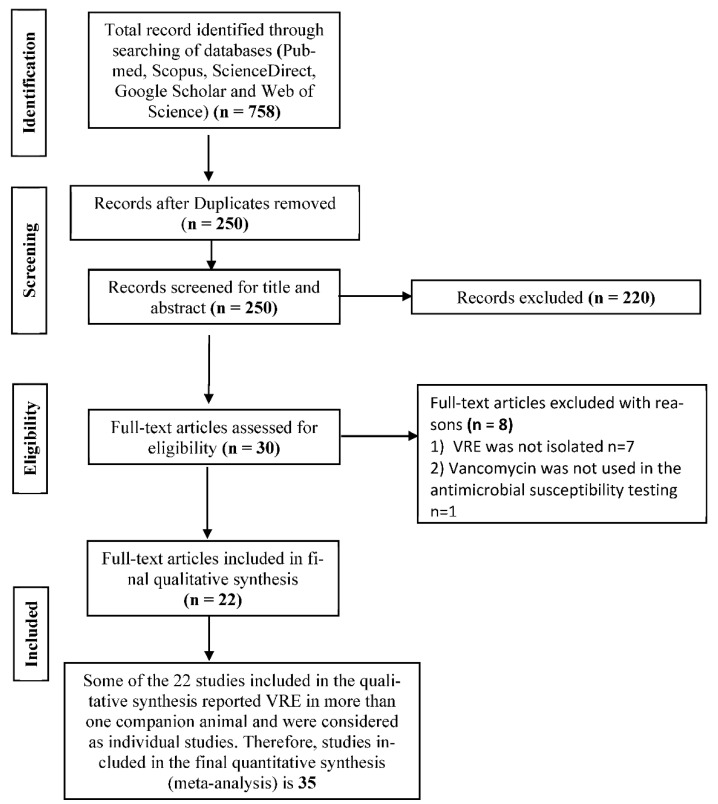
PRISMA flow diagram for the selection of eligible articles included in the study.

A total of 758 studies were found, and 250 were left after duplicates were removed. Of the 250 studies screened for eligibility, 220 were excluded as they did not meet any of the inclusion criteria (studies reporting prevalence of VRE in companion animals, studies in which standard detection methods for VRE were used, and studies reported in English). Thirty full-text articles were assessed for eligibility with eight excluded for lack of sufficient information and the non-use of vancomycin for the antimicrobial susceptibility test (Figure 1). A total of 22 full-text studies were used for qualitative analyses (Figure 1). Of the 4288 isolates tested in the included studies, 1241 were VRE.

However, to get a clearer picture of the prevalence of VRE in companion animals, studies reporting the prevalence in more than one type of companion animals were treated as different studies. Twelve studies reported the prevalence in a single companion animal while the remaining 10 reported the prevalence in more than one companion animal (Table 1). Hence, 35 studies were included in the final meta-analysis. For instance, Devriese et al. (1996) reported the prevalence of VRE in five companion animals (cats, dogs, horses, rabbits, and pheasants), we then considered each companion animal reported in Devrieses et al. (1996) as individual studies denoted by Devriese et al. (1996a,1996b, 1996c, 1996d, and 1996e) (Table 1).

The global distribution of VRE showing country prevalence rates and number of studies reporting VRE is shown in Figure S1.

### 2.2. The Pooled Prevalence of VRE in Companion Animals 

**Figure 2:**
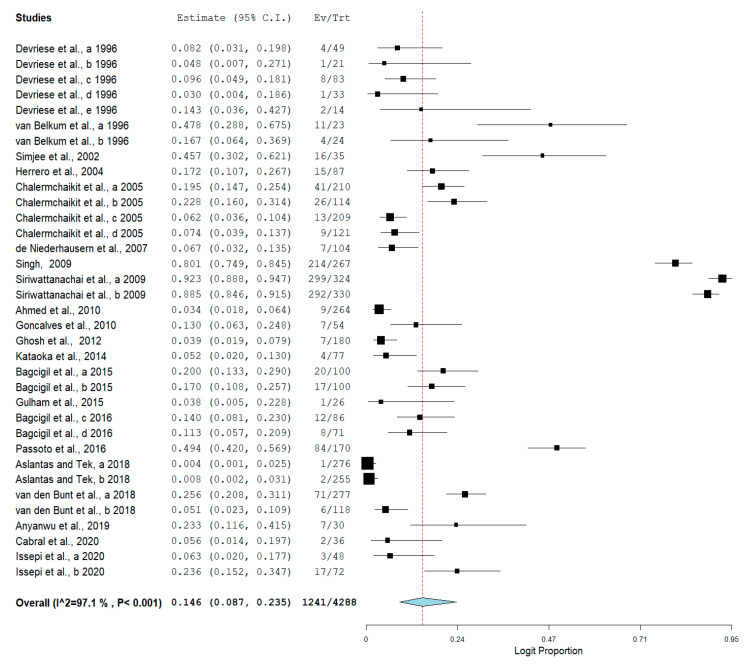
Forest plot showing the pooled prevalence of VRE in companion animals.

**Figure 3:**
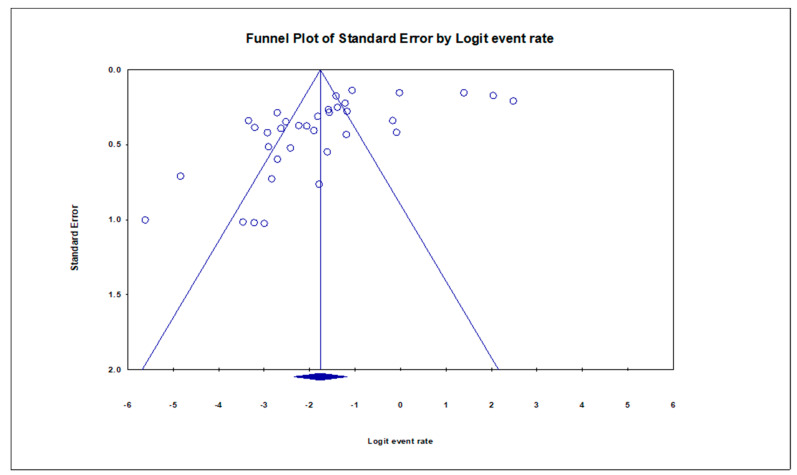
Funnel plot showing publication bias in studies reporting the prevalence of VRE in companion animals.

The pooled prevalence of VRE in companion animals was estimated at 14.6% (95% CI; 8.7–23.5%; *I^2^* = 97.10%; *p* < 0.001) (Figure 2). Random-effects meta-analyses were carried out using the total sample size and number of positives (effect size, standard error of effect size) to estimate the prevalence of VRE in companion animals. Between-study variability was high (*t^2^* = 2.859; heterogeneity *I^2^* = 97.10% with heterogeneity chi-square (*Q*) = 1173.346, degrees of freedom (df) = 34, and *p* < 0.001). No individual study affected the heterogeneity and pooled prevalence of VRE in companion animals as seen in the leave-one-out forest plot that was generated in the sensitivity analysis (Figure S2). More so, publication bias was observed as shown in the asymmetrical funnel plot (Figure 3). Meanwhile, the funnel plot of precision, made no difference (Figure S3). In addition to the funnel plots, Egger’s test was used to confirm the extent of bias (*t*-value = 3.97165; *p* = 0.00036).

### 2.3. Subgroup Meta-Analysis

To identify the possible sources of heterogeneity among studies, as substantial heterogeneity was observed, subgroup analysis was done using the sampling period of the included studies, the countries where the studies were reported, the companion animals in which VRE was detected, the detection method, and sample type collected for VRE detection.

The result of subgroup meta-analysis by sampling period revealed overall large variability in studies reporting the prevalence of VRE (the Higgins *I^2^* statistic = 97.10% with heterogeneity chi-square (*Q*) = 1173.346, degrees of freedom = 34, and *p* < 0.001). However, sampling period was not reported in 11 studies. Sampling period subgroup meta-analysis was thus carried out only on studies with the required information (Table 2). The highest heterogeneity was recorded in studies whose sampling periods were 2014-2015 (*I^2^* = 94.386%) and the heterogeneity of two studies in 2003–2004 was almost moderate (*I^2^* = 62.658%) (Table 2).

The result of subgroup meta-analysis by type of companion animals showed that 15 studies reported the prevalence of VRE in dogs with a pooled prevalence of 18.2% and CI of 9.4–32.5%. This is closely followed by 11 studies reporting the prevalence in cats (12.3%, CI = 3.8–33.1%). Studies reporting the prevalence in horses had the highest heterogeneity (*I^2^* = 98.505%) and accounted for the second highest prevalence (16.9%, CI = 2.2–65.3%) (Table 3).

Further, subgroup meta-analysis by country showed that the majority of the studies were conducted in Turkey (*n = 7*), trailed by Thailand (*n = 6*). Interestingly, India with a single study had the highest prevalence (80.1%, CI = 74.9–84.5%), whereas Turkey with the most studies, had the 4th lowest prevalence (7.2%, CI = 3.4–14.5%). Heterogeneity was highest among studies conducted in Thailand (*I^2^* = 99.040%) which was also trailed by two studies conducted in the United States of America (*I^2^* = 97.137%) (Table 4).

Similarly, the result of subgroup meta-analysis according to sample types revealed that most studies utilized fecal samples (*n = 28*) with a prevalence of 15.9%, and fecal sampling had the highest heterogeneity (*I^2^* = 97.540%)**.** Only five studies utilized rectal swabs with a prevalence of 7.4% and heterogeneity of 92.148% (Table 5).

Finally, the result of subgroup meta-analysis according to the detection method showed that disc diffusion was mostly used (*n = 11*) followed by agar dilution (*n = 7*) and enrichment culture (*n = 7*). Although with two studies, selective culture method had the highest prevalence (30.9%, CI = 9.1–66.4%). Studies that employed agar dilution and disc diffusion had the highest heterogeneity of 98.936% and 97.037%, respectively (Table 6).

### 2.4. Meta-Regression

For every variable included in the study, meta-regression was done separately. The variables were companion animal, sampling period, country, sample type, and detection method. Where the variables had a *p*-value of <0.25, they were used in the multivariable meta-regression analysis. Therefore, all the variables listed above were included in the final analysis. In the multivariate analysis, no data were recorded for the sampling period. For country variables, studies carried out in Italy (*p* = 0.218), Netherlands (*p* = 0.083), Spain (*p* = 0.145), and the United Kingdom (*p* = 0.266) did not contribute to the heterogeneity observed in this study. Pheasants (*p* = <0.001) and rabbit (*p* = <0.001) were the only companion animals that contributed to the study heterogeneity. Studies using rectal swabs (*p* = 0.009) and urine (*p* = 0.012) as sample type were also contributors. Lastly, studies that utilized agar dilution (*p* = <0.001), broth microdilution (*p* = <0.001), and disc diffusion (*p* = <0.001), all contributed to the heterogeneity observed in this study (Table 7).

## 3. Discussion

To our knowledge, this is the first study to use meta-analysis and systematic review to determine the prevalence of VRE in companion animals. The pooled prevalence from this study stems from a thorough analysis of data from scientific publications between 1996–2020 on the prevalence of VRE in companion animals on a global scale. Meta-analysis was carried out on 35 studies. As expected, the literature assessed were heterogeneous, since the review took into account VRE reports from various countries, different sample types, a wide range of sampling periods, and VRE isolated by different methods. This prompted the use of a random effect size model. The high heterogeneity revealed in this research could be attributed to publication bias and small-study effects, since smaller studies sometimes exhibit unconventional, often larger, treatment effects compared to larger ones. A small study with a greater than average impact is more likely to fulfill the statistical significance criterion and could lead to the overestimation of true therapeutic effects. In meta-analysis, assessment of publication bias is vital. This is because not all research findings are published, particularly findings that are deemed unfavorable to a developed protocol or product, or those that would attract only a little interest. Thus, studies that report relatively significant treatment effects are more likely than studies that report more modest treatment effects to be submitted and/or approved for publication. 

Our meta-analysis showed a high variability that suggested that the variability observed was compensated for by other variables in addition to chance. Studies performed in Brazil, India, Japan, Nigeria, Portugal, Thailand, Turkey, and the United States of America led to the elevated heterogeneity seen. Similarly, highly important indicators of heterogeneity between studies were the method of detection, VRE isolated from pheasants and rabbits, and VRE isolated from rectal swabs and urine. There were no prior studies on meta-analysis for comparison, since this is the first study to assess the prevalence of VRE in companion animals.

The highest estimated prevalence of VRE in companion animals was found among dogs. This was expected as dogs and cats are the most owned pets. As stated earlier, the use of avoparcin to improve growth in Europe was banned in food animals in 2006 (EC no. 1831/2003). Before 2006, VRE carriage in dogs increased significantly as seen in the Netherlands where a 48% prevalence was recorded among dogs [18]. Five years after the avoparcin ban, another study found no VRE among 100 dogs [38], suggesting the effectiveness of the avoparcin ban. Subsequently, VRE was documented even more recently in companion animals by Issepi et al. [37] in cats and dogs with a prevalence of 23.6% and 6.25%, respectively. Nosocomial infection in humans and VRE isolated from dogs have been reported to share the same genetic history [19,20,39].

This study shows that VRE has been reported in companion animals in virtually all continents except Australia and Antarctica. Most of the studies (n = 7) were conducted in Europe. Nigeria is the only nation in Africa where a single survey detected VRE in horses [35]. In a systematic review and meta-analysis, Wada et al. [40] reported 25.3% VRE prevalence in Nigeria. Europe has been the hot spot for VRE since it was first reported in humans in the 1980s [7,40,41] and was also the first continent to collectively move for the ban of avoparcin as a growth promoter [42]. It is therefore expected that more studies would be reported from that continent. Veterinary hospitals have reportedly documented the use of broad-spectrum antimicrobial agents for cats and dogs in many European countries, especially the United Kingdom [43]. Further, the law restricting the use of antibiotics in animals in the European Union is not applicable in Turkey, and it is also possible that countries within the European Union get more funding for VRE research.

Interestingly, prior to the restriction on avoparcin use in 1997 [44], 8 studies reported the prevalence of VRE in companion animals, while 27 studies reported the prevalence of VRE in companion animals after the avoparcin ban. As seen in our analysis, the prevalence was gradually rising prior to the ban but continued to fluctuate after the avoparcin ban. The ban on avoparcin or other growth promoters may not have been strictly adhered to and this may be the explanation for the post-avoparcin ban prevalence reported in companion animals. The effect of banning growth promoters, especially in Europe, has led to a decrease in resistant bacteria in food animals [45,46]. Even after the ban, the persistent occurrence of VRE could be due to the link between macrolide and vancomycin used for therapy and growth promotion [47,48]. Zoonotic transmission cannot be ruled out as there is available evidence that shows the transfer of resistant bacteria from companion animals to humans and vice-versa. The direction of the transfer is, however, difficult to prove. In the United States of America for instance, Simjee et al. [19] found a transposon Tn*1546* in a VRE *faecium* isolated from a dog, an observation that has only been described in human clinical VRE. Similarly, Herrero et al. [20] found that VRE of dogs have the same lineage with the VRE that causes nosocomial infections in humans.–

Studies using fecal samples represented the majority of the sample type. Fecal flora is often used to detect resistant genes, since it has been shown to contain a large number of possible pathogens [49,50,51]. Rectal swabs are often used in humans to detect pathogens because they are easy to obtain and easily transported [52]. However, the opposite is the case with animals, as most fecal samples are obtained from the environment. For animals, rectal swabbing is typically difficult and involves a great deal of planning and expertise [53]. A good technique for sample collection is that which reduces DNA lyses or alteration for molecular studies, as many studies have shown how important it is for sample collection and storage, library preparation, and DNA extraction using a standardized procedure [54,55,56]. It has also been shown that regardless of the condition (fresh or frozen) of fecal or rectal samples used, there is no significant difference in the makeup of intestinal microflora [54,55]. Artim et al. [57] asserted that there is an increasing awareness of differences in the makeup of intestinal microbiota in distinct compartments of the gastrointestinal tract. However, conflicting opinions regarding the composition of the microbiome in either a sick or a healthy animal tend to be present. The dissimilarity between the microbiome in fecal samples of healthy and sick Rhesus macaques was reported by McKenna et al. [58], while Yasuda et al. [59] reported a similarity in the microbiome from fecal samples. However, in humans, Bassis et al. [52] demonstrated in their study that there are no variations in microbiota between the feces and rectal swab samples of the same individual.

For the detection of VRE from rectal swabs or in stool samples, it is imperative to state that no medium is the gold standard, although several have been used [60,61]. The broth microdilution method is more sensitive than the disc diffusion for the detection of VRE [62,63], although it requires a full-day incubation to detect VRE with low-level resistance. The use of broth enrichment for VRE detection has also been promoted in several studies [60,61].

The strength of our study is that we considered VRE globally in studies with sampling periods as far back as 1995. We also critically considered variation in methods, sample sources, and companion animal species. The pooled prevalence of VRE at species levels as well as the pooled prevalence of the resistant genes could not be estimated by our research.

## 4. Materials and Methods

### 4.1. Study Design and Protocol

The Preferred Reporting Items for Systematic Reviews and Meta-Analysis Protocol (PRISMA-P 2015) guidelines [64] was used as the checklist for this study (Supplementary File S1).

### 4.2. Literature Review

To ensure no other meta-analysis on the prevalence of VRE in companion animals exists or is ongoing, the PROSPERO database and database of abstracts of reviews of effects (DARE) (http://www.library.UCSF.edu) were searched. This was then followed by searching MedLib, PubMed, Web of Science, Scopus, and Google Scholar for published studies about the prevalence of VRE in companion animals. All these databases were searched using the search strategy; “vre”[All Fields] AND (“pets”[MeSH Terms] OR “pets”[All Fields] OR (“companion”[All Fields] AND “animals”[All Fields]) OR “companion animals”[All Fields]), “vre”[All Fields] AND (“dogs”[MeSH Terms] OR “dogs”[All Fields]) AND “cats”[All Fields], “vre”[All Fields] AND (“cats”[MeSH Terms] OR “cats”[All Fields]), “vre”[All Fields] AND (“horses”[All Fields] OR “horses”[MeSH Terms] OR “horses”[All Fields] OR “horse”[All Fields] OR “equidae”[MeSH Terms] OR “equidae”[All Fields]), “vre”[All Fields] AND (“rabbits”[All Fields] OR “rabbits”[MeSH Terms] OR “rabbits”[All Fields] OR “rabbit”[All Fields]), “VRE”[All Fields] AND “ostrich”[All Fields]. In addition, references and titles from included articles were utilized as a supplementary search tool. Two authors carried out the search to minimize bias.

### 4.3. Inclusion and Exclusion Criteria for Studies

All cross-sectional or cohort studies that reported the prevalence of VRE isolates or numbers of VRE and total enterococci isolates in companion animals were included. For this study, we defined companion animals as dogs, cats, parrots, pheasants, horses, ostriches, and rabbits used as pets and not for consumption. In addition, studies published or reported in English in which the standard method (method approved for use according to the Clinical and Laboratory Standards Institute (CLSI) guidelines) was used to detect VRE were included.

Studies with insufficient information, studies on antimicrobial susceptibility tests other than vancomycin, studies not reporting enterococcal isolates separately (no population denominator), reviews, comments and duplications, case report studies, and studies that did not report the prevalence of VRE in companion animals were excluded.

### 4.4. Data Extraction

Identification of studies was done based on our exclusion criteria and studies to be included were scrutinized in three steps: title, abstract, and full text. The first author’s name, publication year, sampling period, type of companion animal, study country, number of cases involved in the studies, detection method, sample types, sample size, and the prevalence of VRE infections were extracted from the manuscripts. Two independent reviewers extracted all data from the included articles, and the results were reviewed by a third reviewer. Discrepancies between the reviewers were resolved by a consensus.

### 4.5. Study Quality Assessment

The quality of the included studies was evaluated by the Joanna Briggs Institute (JBI) critical appraisal checklist for prevalence data [65] (Supplementary File S2). This appraisal checklist contains nine items that assess (1) appropriate sampling frame, (2) proper sampling technique, (3) adequate sample size, (4) study subject and setting description, (5) sufficient data analysis, (6) use of valid methods for the identified conditions, (7) valid measurement for all participants, (8) using appropriate statistical analysis, and (9) adequate response rate. Each item is graded as yes, no, unclear or not applicable. A score of 1 was allotted for the ‘yes’ response, while 0 scores were provided for ‘no’ and ‘unclear’ responses. Finally, the mean score was calculated for each article. Then, studies with scores below and above the mean were characterized as poor and good quality respectively [65]. Studies were included in the analysis if consensus was reached among the two reviewers. The quality of the 35 included studies is given in (Supplementary File S3).

### 4.6. Data Analysis

Prevalence of VRE in companion animals was calculated, and subgroup analyses were done according to the sampling period, study country, the companion animal, sample type, and detection method. Where the prevalence was not reported by a study, they were back-calculated. Because studies were carried out in diverse settings and populations, heterogeneity was expected and the random-effects model was thus used in determining the pooled prevalence of VRE in companion animals using the DerSimonian and Laird method of meta-analysis [66,67].

### 4.7. Bias and Heterogeneity Analysis

The study country, sampling period, sample type, companion animal, and detection method were used to assess the within-study biases. Small study effects or bias were examined by funnel plots and precision funnel plots. The heterogeneities of study-level estimates were assessed by Cochran’s *Q* test. Non-significant heterogeneity was accepted if the ratio of *Q* and the degrees of freedom (Q/df) was less than one. The percentage of the variation in prevalence estimates attributable to heterogeneity was measured by the inverse variance index (*I^2^*), and *I^2^* values of 25%, 50%, and 75% were considered low, moderate, and high heterogeneity, respectively [68]. In our meta-analysis of VRE in companion animals, the *I^2^* value was high (97.10%), where >75% is an indication of significant heterogeneity, which prompted the use of random-effects model at 95% CI instead of the fixed-effects model. The sources of heterogeneity were analyzed using the sensitivity analysis (leave-one-out meta-analysis), subgroup analysis, and meta-regression. Meta-analysis was performed using OpenMeta Analyst software [68] and Comprehensive meta-analysis version 2 [69].

## 5. Conclusions

There is ample evidence suggesting the existence of drug-resistant bacteria in companion animals as well as their transmissibility to humans. In this study, a systematic review and meta-analysis of studies reporting the prevalence of VRE in companion animals around the globe was conducted, and a pooled prevalence estimated at 14.6% was obtained. However, due to the relatively high heterogeneity observed, it is difficult to conclude that this estimate represents the true point estimate. Nevertheless, we believe the estimate provides a good idea of the prevalence of VRE in companion animals. With the advent of resistant bacterial strains to existing drugs, including vancomycin, there is a need to explore newer antimicrobials in veterinary medicine. Routine monitoring of VRE in companion animals would help inform policymaking for proper control.

## Figures and Tables

**Table 1:** Characteristics of included studies reporting the prevalence of vancomycin-resistant *Enterococcus* (VRE) in companion animals.

S/No	Author, Publication Year	Sampling Period	Country	Companion Animal	Sample Size	Number Positive	Prevalence (%)	Detection Method	Sample Type	Study Design
1a	Devriese et al., 1996 [17]	1995	Belgium	Dog	49	4	8.16	Enrichment culture	Fecal	Cross-sectional
1b	Devriese et al., 1996 [17]	1995	Belgium	Cat	21	1	4.76	Enrichment culture	Fecal	Cross-sectional
1c	Devriese et al., 1996 [17]	1995	Belgium	Horse	83	8	9.64	Enrichment culture	Fecal	Cross-sectional
1d	Devriese et al., 1996 [17]	1995	Belgium	Rabbit	33	1	3.03	Enrichment culture	Fecal	Cross-sectional
1e	Devriese et al., 1996 [17]	1995	Belgium	Pheasants	14	2	14.29	Enrichment culture	Fecal	Cross-sectional
2a	van Belkum et al., 1996 [18]	1996	Netherlands	Dog	23	11	48	Selective culture	Rectal swab	Cross-sectional
2b	van Belkum et al., 1996 [18]	1996	Netherlands	Cat	24	4	16	Selective culture	Rectal swab	Cross-sectional
3	Simjee et al., 2002 [19]	1996–1998	United States of America	Dog	35	16	45.71	Broth microdilution	Urine	Cross-sectional
4	Herrero et al., 2004 [20]	1998–2003	Spain	Dog	87	15	17.24	Sensititre system	Fecal	Cross-sectional
5a	Chalermchaikit et al., 2005 [21]	-	Thailand	Dog	210	41	19.5	Agar dilution	Fecal	Cross-sectional
5b	Chalermchaikit et al., 2005 [21]	-	Thailand	Cat	114	26	22.8	Agar dilution	Fecal	Cross-sectional
6a	Chalermchaikit et al., 2005 [21]	-	Thailand	Dog	209	13	6.22	Agar dilution	Fecal	Cross-sectional
6b	Chalermchaikit et al., 2005 [21]	-	Thailand	Cat	121	9	7.44	Agar dilution	Fecal	Cross-sectional
7	de Niederhausern et al., 2007 [22]	2005	Italy	Horse	104	7	6.73	Agar dilution	Fecal	Cross-sectional
8	Singh, 2009 [23]	2008	India	Horse	267	214	80.2	Disc diffusion	Fecal	Cross-sectional
9a	Siriwattanachai et al., 2009 [24]	2003–2004	Thailand	Dog	324	299	92.28	Agar dilution	Fecal	Cross-sectional
9b	Siriwattanachai et al., 2009 [24]	2003–2004	Thailand	Cat	330	292	88.48	Agar dilution	Fecal	Cross-sectional
10	Ahmed et al., 2011 [25]	2010	United Kingdom	Horse	264	9	3.41	Enrichment agar	Fecal	Cross-sectional
11	Goncalves et al., 2010 [26]	-	Portugal	Ostrich	54	7	13.0	Disc diffusion	Fecal	Cross-sectional
12	Ghosh et al., 2012 [27]	-	United States of America	Cat	180	7	3.89	Disc diffusion	Fecal	Cross-sectional
13	Kataoka et al., 2014 [28]	2011–2012	Japan	Dog	77	4	5.19	Broth microdilution	Fecal	Cross-sectional
14a	Bagcigil et al., 2015 [29]	-	Turkey	Dog	100	20	20.0	Broth microdilution	Fecal	Cross-sectional
14b	Bagcigil et al., 2015 [29]	-	Turkey	Cat	100	17	17.0	Broth microdilution	Fecal	Cross-sectional
15	Gulhan et al., 2015 [30]	-	Turkey	Dog	26	1	3.85	Disc diffusion	Fecal	Cross-sectional
16a	Bagcigil et al., 2016 [31]	2015	Turkey	Dog	86	12	13.95	Disc diffusion	Fecal	Cross-sectional
16b	Bagcigil et al., 2016 [31]	2015	Turkey	Cat	71	8	11.27	Disc diffusion	Fecal	Cross-sectional
17	Pasotto et al., 2016 [32]	-	Italy	Dog	170	84	49.41	Disc diffusion	Fecal	Cross-sectional
18a	Aslantas and Tek, 2019 [33]	2018	Turkey	Dog	276	1	0.13	Disc diffusion	Rectal swab	Cross-sectional
18b	Aslantas and Tek, 2019 [33]	2018	Turkey	Cat	255	2	0.8	Disc diffusion	Rectal swab	Cross-sectional
19a	van den Bunt et al., 2018 [34]	2014–2015	Netherlands	Dog	277	71	25.63	Enrichment culture	Fecal	Cross-sectional
19b	van den Bunt et al., 2018 [34]	2014–2015	Netherlands	Cat	118	6	5.08	Enrichment culture	Fecal	Cross-sectional
20	Anyanwu et al., 2019 [35]	2018	Nigeria	Horse	30	7	23.3	Disc diffusion	Rectal swab	Cross-sectional
21	Cabral et al., 2020 [36]	-	Brazil	Parrots	36	2	5.5	Disc diffusion	Cloacal swab	Cross-sectional
22a	Issepi et al., 2020 [37]	2017	Italy	Dog	48	3	6.25	Broth microdilution	Fecal	Cross-sectional
22b	Issepi et al., 2020 [37]	2017	Italy	Cat	72	17	23.6	Broth microdilution	Fecal	Cross-sectional

**Table 2:** Subgroup analysis for comparison of prevalence of VRE in companion animals across sampling periods.

Sampling Period	Number of Studies	Prevalence (%)	95% CI	*I^2^* (%)	*Q*	Heterogeneity Test	
						DF	*p*
1995	5	8.6	5.3–13.6	0.00	2.245	4	0.691
1996	2	30.9	9.1–66.4	79.539	4.887	1	0.027
1996–1998	1	45.7	30.2–62.1	0.00	0.00	0	1.00
1998–2003	1	17.2	10.7–26.7	0.00	0.00	0	1.00
2003–2004	2	90.4	86.0–93.6	62.658	2.678	1	0.102
2005	1	6.7	3.2–13.5	0.00	0.00	0	1.00
2008	1	80.1	74.9–84.5	0.00	0.00	0	1.00
2010	1	3.4	1.8–6.4	0.00	0.00	0	1.00
2011-2012	1	5.2	2.0–13.0	0.00	0.00	0	1.00
2014-2015	2	12.4	2.2–46.7	94.386	17.814	1	0.00
2015	2	12.8	8.4–19.0	0.00	0.252	1	0.616
2017	2	13.6	3.4–41.2	81.616	5.440	1	0.020
2018	3	2.2	0.1–30.8	93.266	29.701	2	0.00

**Table 3:** Subgroup analysis for comparison of prevalence of VRE in companion animals according to the type of companion animal.

Companion Animal	Number of Studies	Prevalence (%)	95% CI	*I^2^* (%)	*Q*	Heterogeneity Test	
						DF	*p*
Cat	11	12.3	3.8–33.1	97.654	424.639	10	0.00
Dog	15	18.2	9.4–32.5	96.745	430.158	14	0.00
Horse	5	16.9	2.2–65.3	98.505	267.595	4	0.00
Ostrich	1	13.0	6.3–24.8	0.00	0.00	0	1.00
Parrot	1	5.6	1.4–19.7	0.00	0.00	0	1.00
Pheasant	1	14.3	3.6–42.7	0.00	0.00	0	1.00
Rabbit	1	3.0	0.4–18.6	0.00	0.00	0	1.00

**Table 4:** Subgroup analysis for comparison of prevalence of VRE in companion animals according to country.

Country	Number of Studies	Prevalence (%)	95% CI	*I^2^* (%)	*Q*	Heterogeneity Test	
						DF	*p*
Belgium	5	8.6	5.3–13.6	0.00	2.245	4	0.691
Brazil	1	5.6	1.4–19.7	0.00	0.00	0	1.00
India	1	80.1	74.9–84.5	0.00	0.00	0	1.00
Italy	4	17.2	5.3–43.4	94.801	57.703	3	0.00
Japan	1	5.2	2.0–13.0	0.00	0.00	0	1.00
Netherlands	4	19.8	8.3–40.2	88.249	25.529	3	0.00
Nigeria	1	23.3	11.6–41.5	0.00	0.00	0	1.00
Portugal	1	13.0	6.3–24.8	0.00	0.00	0	1.00
Spain	1	17.2	10.7–26.7	0.00	0.00	0	1.00
Thailand	6	36.6	8.8–77.5	99.040	520.613	5	0.00
Turkey	7	7.2	3.4–14.5	84.383	38.421	6	0.00
United Kingdom	1	3.4	1.8–6.4	0.00	0.00	0	1.00
United States of America	2	15.7	0.9–78.4	97.137	34.934	1	0.00

**Table 5:** Subgroup analysis for comparison of prevalence of VRE in companion animals according to sample types.

Sample Types	Number of Studies	Prevalence (%)	95% CI	*I^2^* (%)	*Q*	Heterogeneity Test	
						DF	*p*
Cloacal swab	1	5.5	1.4–19.7	0.00	0.00	0	1.00
Fecal	28	15.9	9.0–26.6	97.540	1097.423	27	0.00
Rectal swab	5	7.4	1.4–31.7	92.148	50.941	4	0.00
Urine	1	45.7	30.2–62.1	0.00	0.00	0	1.00

**Table 6:** Subgroup analysis for comparison of prevalence of VRE in companion animals according to detection methods.

Detection Method	Number of Studies	Prevalence (%)	95% CI	*I^2^* (%)	*Q*	Heterogeneity Test	
						DF	*p*
Agar dilution	7	30.1	7.5–69.5	98.936	563.712	6	0.00
Broth microdilution	6	17.5	10.1–28.6	81.876	27.588	5	0.00
Disc diffusion	11	10.2	3.5–26.2	97.037	337.479	10	0.00
Enrichment culture	1	3.4	1.8–6.4	0.00	0.00	0	1.00
Enrichment culture	7	9.3	4.3–18.8	82.647	34.576	6	0.00
Selective culture	2	30.9	9.1–66.4	79.539	4.887	1	0.027
Sensititre system	1	17.2	10.7–26.7	0.00	0.00	0	1.00

**Table 7:** Final multivariable meta-regression model.

Variable	Coefficient	95% CI	*p*-Value
**Country**			
Belgium	Reference		
Brazil	−0.538	−0.747–−0.329	<0.001
India	0.705	0.587–0.823	<0.001
Italy	−0.099	−0.257–0.059	0.218
Japan	−0.188	−0.352–−0.024	0.025
Netherlands	0.081	−0.010–0.172	0.083
Nigeria	−0.359	−0.664–−0.053	0.021
Portugal	−0.464	−0.679–−0.249	<0.001
Spain	0.093	−0.032–0.218	0.145
Thailand	−0.527	−0.879–−0.175	0.003
Turkey	−0.555	−0.804–−0.306	<0.001
United Kingdom	−0.062	−0.172–0.047	0.266
United States of America	−0.526	−0.723–−0.329	<0.001
**Companion Animal**			
Dog	Reference		
Cat	−0.029	−0.067–0.009	0.141
Horse	0.017	−0.099–0.133	0.772
Ostrich	0.064	−0.144–0.271	0.548
Parrot	−0.049	−0.162–0.064	0.397
Pheasant	0.496	0.253–0.738	<0.001
Rabbit	0.743	0.513–0.973	<0.001
**Sample Type**			
Fecal	References		
Cloacal swab	0.070	−0.127–0.267	0.485
Rectal swab	0.161	0.041–0.281	0.009
Urine	−0.357	−0.637–−0.078	0.012
**Detection Method**			
Enrichment culture			
Agar dilution	0.514	0.345–0.683	<0.001
Broth microdilution	1.296	1.115–1.476	<0.001
Disc diffusion	0.616	0.368–0.864	<0.001
Constant	0.079	0.004–0.154	0.039

## Data Availability

The datasets used and/or analyzed during the current study are included in the manuscript.

## Supplementary Materials

The following are available online at https://www.mdpi.com/2079-6382/10/2/138/s1, Figure S1. Spatial distribution and number of studies of VRE in Companion animals based on data extracted from eligible studies; Figure S2. Leave-one-out forest plot of VRE in companion animals; Figure S3. Funnel plot of precision showing publication bias in studies reporting the prevalence of VRE in companion animals. File S1 PRISMA 2009 Checklist; File S2 Checklist for Prevalence Studies; File S3 Quality of included studies by JBI critical appraisal checklist for studies reporting prevalence data.
