# Supplementary material for: Prevalence of Vancomycin-Resistant Enterococcus (VRE) in Companion Animals: The First Meta-Analysis and Systematic Review
Source: Antibiotics (Basel). 2021 Jan 31;10(2):138. doi: 10.3390/antibiotics10020138 (PMC7911405; doi:10.3390/antibiotics10020138)
Supplement: Supplementary file 1 [file antibiotics-10-00138-s001.zip › antibiotics-1071922 sup figure.pdf]

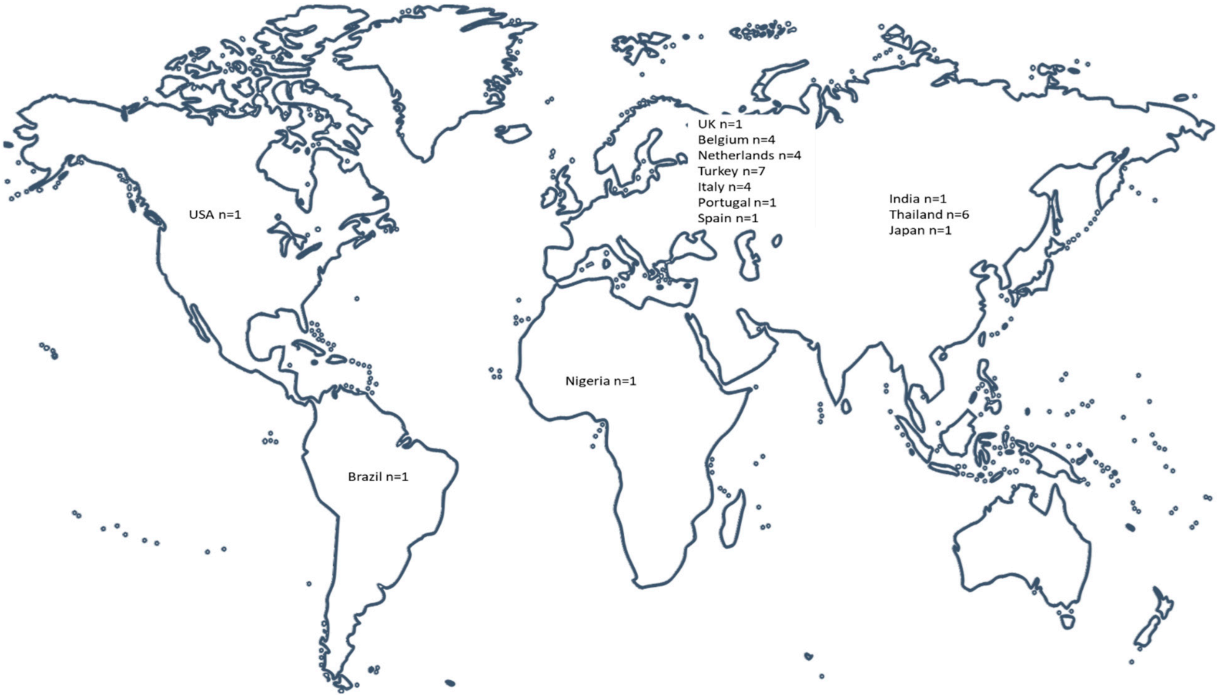

Figure S1. Spatial distribution and number of studies of VRE in Companion animals based on data extracted from eligible studies.

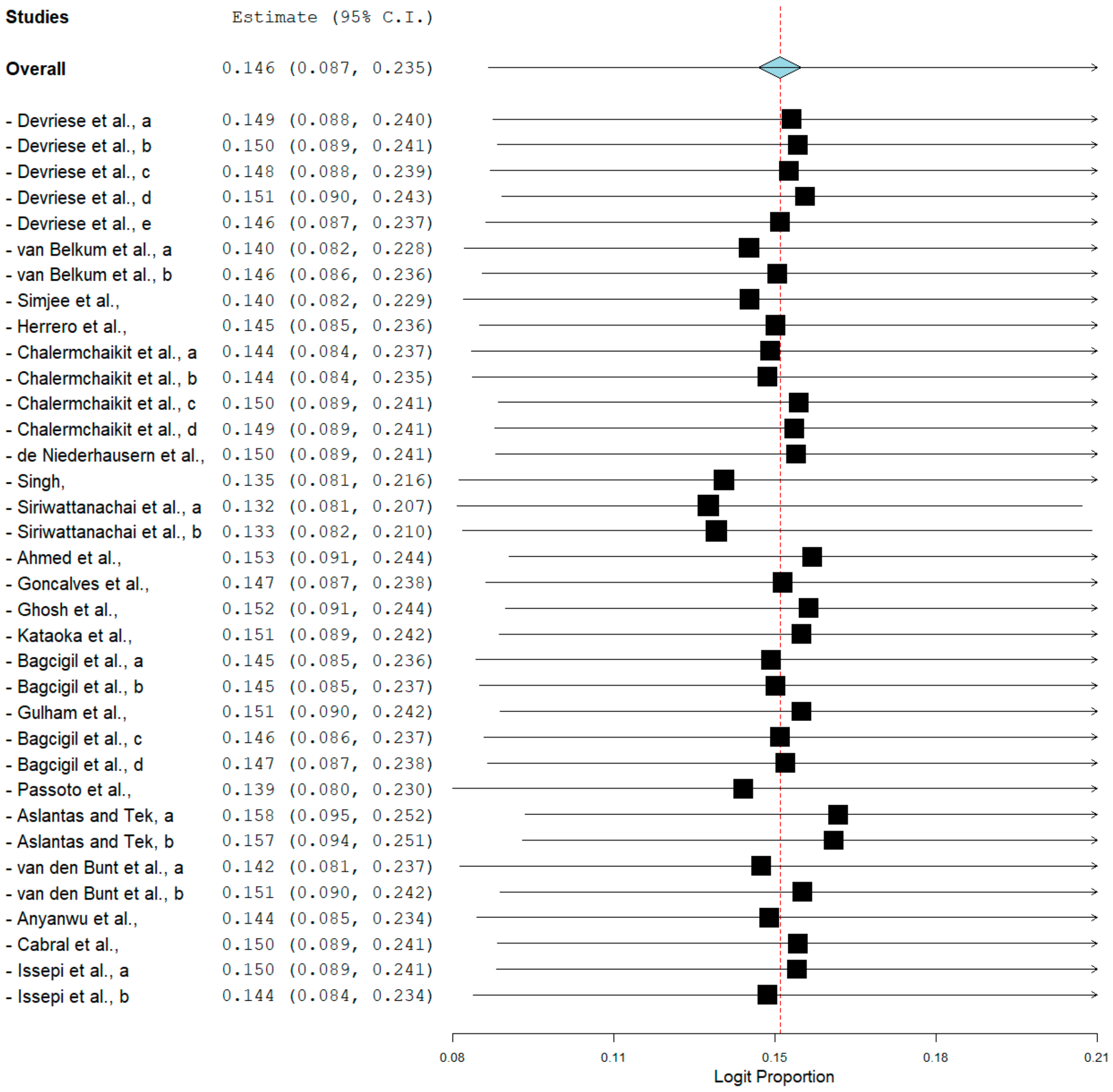

Figure S2. Leave-one-out forest plot of VRE in companion animals.

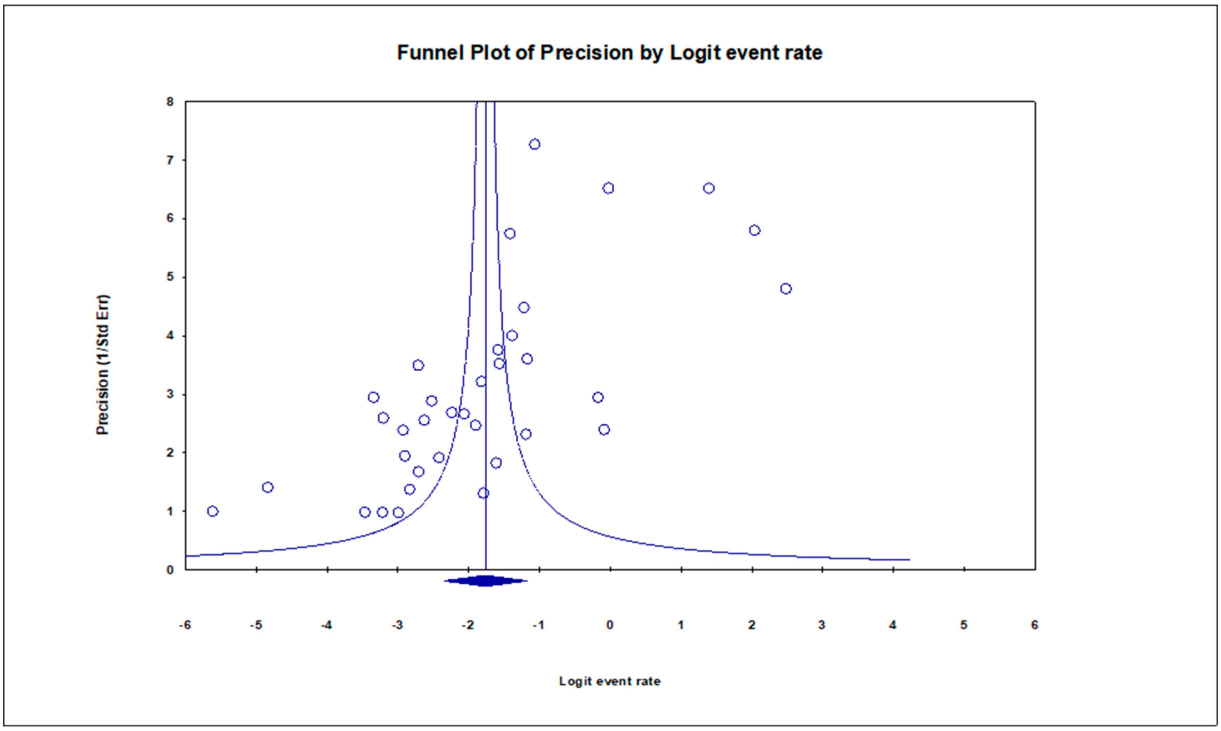

Figure S3. Funnel plot of precision showing publication bias in studies reporting the prevalence of VRE in companion animals.
