# Supplementary material for: Prevalence of Vancomycin-Resistant Enterococcus (VRE) in Companion Animals: The First Meta-Analysis and Systematic Review
Source: Antibiotics (Basel). 2021 Jan 31;10(2):138. doi: 10.3390/antibiotics10020138 (PMC7911405; doi:10.3390/antibiotics10020138)
Supplement: Supplementary file 1 [file antibiotics-10-00138-s001.zip › Supplementary File S3 VRE in CA.pdf]

Quality of included studies by JBI critical appraisal checklist for studies reporting prevalence data

Study name		a									Overall
		1	2	3	4	5	6	7	8	9	
1	Devriese et al., 1996	Yes	**No**	Yes	Yes	Yes	Yes	Yes	Yes	Unclear	7
2	van Belkum et al., 1996	Yes	**No**	Yes	Yes	Yes	Yes	Yes	Yes	Yes	8
3	Simjee et al., 2002	Yes	**No**	Yes	Yes	Yes	Yes	Yes	Yes	Yes	8
4	Herrero et al., 2004	Yes	**No**	Yes	Yes	Yes	Yes	Yes	Yes	Yes	8
5	Chalermchaikit et al., 2005	Yes	**No**	Yes	Yes	Yes	Yes	Yes	Yes	Yes	8
6	Chalermchaikit et al., 2005	Yes	**No**	Yes	Yes	Yes	Yes	Yes	Yes	Yes	8
7	de Niederhausern et al., 2007	Yes	**No**	Yes	Yes	Yes	Yes	Yes	Yes	Yes	8
8	Singh, 2009	Yes	**No**	Yes	Yes	Yes	Yes	Yes	Yes	Yes	8
9	Siriwattanachai et al., 2009	Yes	**No**	Yes	Yes	Yes	Yes	Yes	Yes	Yes	8
10	Ahmed et al., 2011	Yes	**No**	Yes	Yes	Yes	Yes	Yes	Yes	Yes	8
11	Goncalves et al., 2010	Yes	**No**	Yes	Yes	Yes	Yes	Yes	Yes	Yes	8
12	Ghosh et al., 2012	Yes	**No**	Yes	Yes	Yes	Yes	Yes	Yes	Yes	8
13	Kataoka et al., 2014	Yes	**No**	Yes	Yes	Yes	Yes	Yes	Yes	Yes	8
14	Bagcigil et al., 2015	Yes	**No**	Yes	Yes	Yes	Yes	Yes	Yes	Yes	8
15	Gulhan et al., 2015	Yes	**No**	Yes	Yes	Yes	Yes	Yes	Yes	Yes	8
16	Bagcigil et al., 2016	Yes	**No**	Yes	Yes	Yes	Yes	Yes	Yes	Yes	8
17	Pasotto et al., 2016	Yes	**No**	Yes	Yes	Yes	Yes	Yes	Yes	Yes	8
18	Aslantas and Tek, 2019	Yes	**No**	Yes	Yes	Yes	Yes	Yes	Yes	Yes	8
19	van den Bunt et al., 2018	Yes	**No**	Yes	Yes	Yes	Yes	Yes	Yes	Yes	8
20	Anyanwu et al., 2019	Yes	**No**	Yes	Yes	Yes	Yes	Yes	Yes	Yes	8
21	Cabral et al., 2020	Yes	**No**	Yes	Yes	Yes	Yes	Yes	Yes	Yes	8
22	Issepi et al., 2020	Yes	**No**	Yes	Yes	Yes	Yes	Yes	Yes	Yes	8

* **1.** Appropriate sampling frame to address the target population, **2.** Appropriate sampling way of study participants, **3.** Adequate sample size, **4.** Detail description of study participants and settings, **5.** Data analysis with sufficient coverage of the identified sample, **6.** Use of valid methods to identify the condition, **7.** Standard, reliable way of measurement of condition for all participants, **8.** Availability of appropriate statistical analysis, **9.** Adequate response rate and management of low response rate
